# Pyrimethaminium 2-{[4-(tri­fluoro­meth­yl)phen­yl]sulfan­yl}benzoate dimethyl sulfoxide monosolvate

**DOI:** 10.1107/S1600536814010411

**Published:** 2014-05-17

**Authors:** Thammarse S. Yamuna, Manpreet Kaur, Jerry P. Jasinski, H.S. Yathirajan

**Affiliations:** a Department of Studies in Chemistry, University of Mysore, Manasagangotri, Mysore 570 006, India; b Department of Chemistry, Keene State College, 229 Main Street, Keene, NH 03435-2001, USA

## Abstract

In the cation of the title solvated mol­ecular salt, C_12_H_14_ClN_4_^+^·C_14_H_8_F_3_O_2_S^−^·C_2_H_6_OS [systematic name of the cation: 2,4-di­amino-5-(4-chloro­phen­yl)-6-ethyl­pyrimidin-1-ium], the dihedral angle between the planes of the pyrimidinium and 4-chloro­phenyl rings is 77.2 (5)°. In the anion, the planes of the benzene rings are twisted with respect to each other by 71.5 (5)°. Disorder was modelled for the dimethyl sulfoxide solvent mol­ecule over two set of sites in a 0.7487 (13):0.2513 (13) ratio. In the crystal, the cations are linked by inversion-generated pairs of N—H⋯N hydrogen bonds, with an *R*_2_^2^(8) graph-set motif. The cation donates two N—H⋯O hydrogen bonds to the anion, also generating an *R*_2_^2^(8) loop. These inter­actions, along with cation–solvent N—H⋯O hydrogen bonds, and cation–anion C—H⋯F, solvent–anion C—H⋯O and C—H⋯F inter­actions, result in a three-dimensional network.

## Related literature   

For background to pyrimethamine, see: Kraut & Matthews (1987 ▶); Zuccotto *et al.* (1998 ▶). For supra­molecular synthons, see: Desiraju (1995 ▶). For related structures, see: Balasubramani *et al.* (2005 ▶); Devi *et al.* (2006 ▶, 2007 ▶); Ebenezer & Mu­thiah (2010 ▶); Subashini *et al.* (2007 ▶); Thanigaimani *et al.* (2009 ▶); Yamuna *et al.* (2013 ▶).
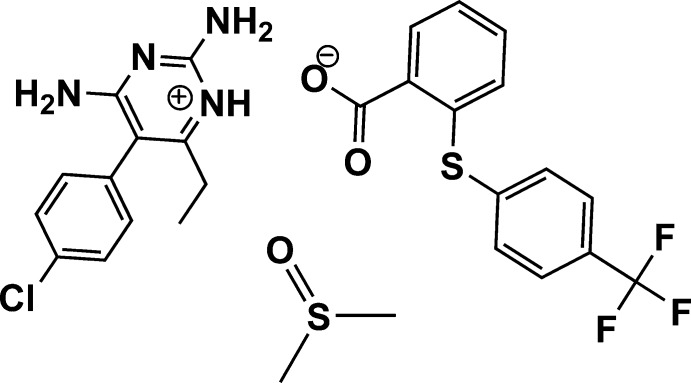


## Experimental   

### 

#### Crystal data  

C_12_H_14_ClN_4_^+^·C_14_H_8_F_3_O_2_S^−^·C_2_H_6_OS
*M* *_r_* = 625.11
Monoclinic, 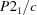

*a* = 12.7422 (3) Å
*b* = 22.2773 (3) Å
*c* = 11.1761 (3) Å
β = 114.014 (3)°
*V* = 2897.88 (12) Å^3^
*Z* = 4
Cu *K*α radiation
μ = 3.01 mm^−1^
*T* = 173 K
0.36 × 0.18 × 0.06 mm

#### Data collection  

Agilent Eos Gemini diffractometer
Absorption correction: multi-scan (*CrysAlis RED*; Agilent, 2012 ▶) *T*_min_ = 0.374, *T*_max_ = 1.000
19462 measured reflections
5571 independent reflections
4889 reflections with *I* > 2σ(*I*)
*R*_int_ = 0.046

#### Refinement  

*R*[*F* ^2^ > 2σ(*F* ^2^)] = 0.049
*wR*(*F* ^2^) = 0.138
*S* = 1.02
5571 reflections
389 parameters
54 restraints
H-atom parameters constrained
Δρ_max_ = 0.72 e Å^−3^
Δρ_min_ = −0.41 e Å^−3^

### 

Data collection: *CrysAlis PRO* (Agilent, 2012 ▶); cell refinement: *CrysAlis PRO*; data reduction: *CrysAlis RED* (Agilent, 2012 ▶); program(s) used to solve structure: *SUPERFLIP* (Palatinus & Chapuis, 2007 ▶); program(s) used to refine structure: *SHELXL2012* (Sheldrick, 2008 ▶); molecular graphics: *OLEX2* (Dolomanov *et al.*, 2009 ▶); software used to prepare material for publication: *OLEX2*.

## Supplementary Material

Crystal structure: contains datablock(s) I. DOI: 10.1107/S1600536814010411/hb7223sup1.cif

Structure factors: contains datablock(s) I. DOI: 10.1107/S1600536814010411/hb7223Isup2.hkl

Supporting information file. DOI: 10.1107/S1600536814010411/hb7223Isup3.cml

CCDC reference: 1001667

Additional supporting information:  crystallographic information; 3D view; checkCIF report

## Figures and Tables

**Table 1:** Hydrogen-bond geometry (Å, °)

*D*—H⋯*A*	*D*—H	H⋯*A*	*D*⋯*A*	*D*—H⋯*A*
N1—H1⋯O1	0.88	1.79	2.674 (2)	178
N3—H3*A*⋯O1*SA* ^i^	0.88	2.20	3.046 (6)	162
N3—H3*A*⋯O1*SB* ^i^	0.88	2.13	2.97 (2)	161
N3—H3*B*⋯O2	0.88	1.93	2.809 (2)	176
N4—H4*A*⋯N2^ii^	0.88	2.15	3.030 (2)	175
N4—H4*B*⋯O1*SA* ^iii^	0.88	2.25	2.962 (4)	138
N4—H4*B*⋯O1*SB* ^iii^	0.88	2.06	2.740 (16)	133
C12—H12⋯F3^iii^	0.95	2.57	3.444 (2)	153
C2*SA*—H2*SB*⋯O2^iv^	0.98	2.44	3.376 (6)	160
C2*SB*—H2*SE*⋯F1^v^	0.98	2.55	3.16 (3)	120
C2*SB*—H2*SF*⋯O2^iv^	0.98	2.47	3.21 (2)	132

Symmetry codes: (i) 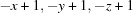; (ii) 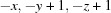; (iii) 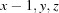; (iv) 
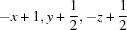
; (v) 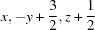.

**Fig. 1.**
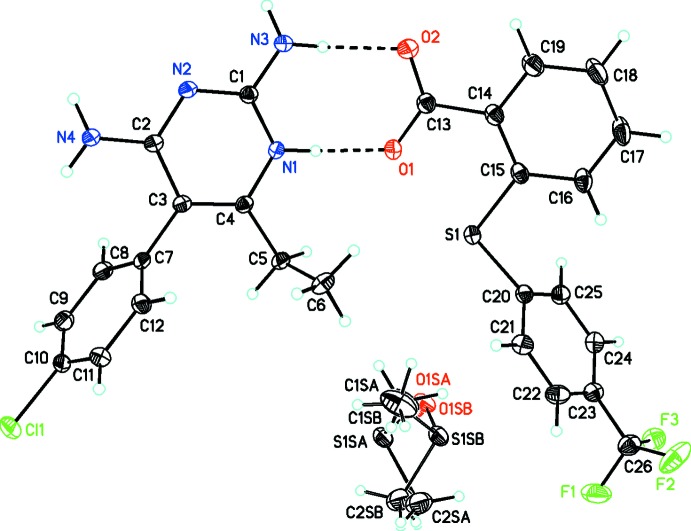
ORTEP drawing of (I) showing 30% probability displacement ellipsoids. Dashed lines indicate N—H···O hydrogen bonds within the asymmetric unit forming R22(8) graph-set ring motifs.

**Fig. 2.**
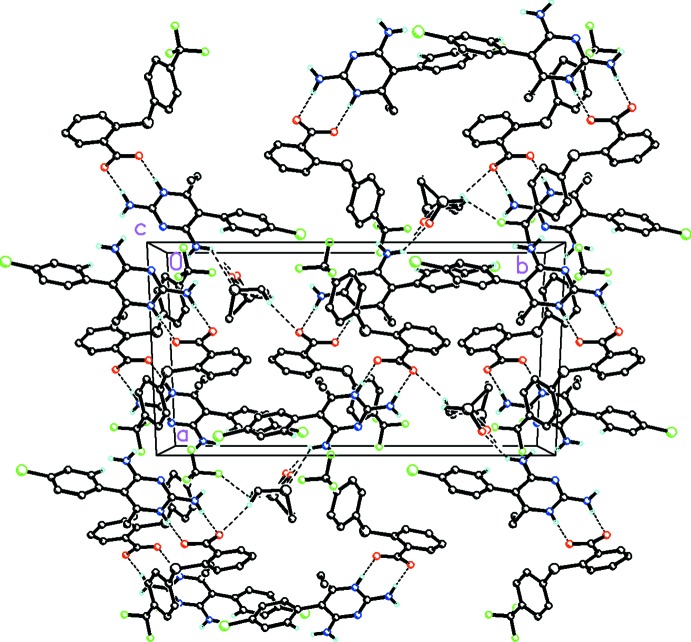
Molecular packing for (I) viewed along the c axis. Dashed lines indicate cation-anion N—H···O hydrogen bonds (forming R22(8) graph-set ring motifs) along with cation-cation N—H···N hydrogen bonds, cation-solvate N—H···O hydrogen bonds and weak cation-anion C—H···F, and solvate-anion C—H···O, C—H···F interactions.

## supplementary crystallographic information

### 1. Comment

Pyrimethamine (trade name Daraprim; {5-(4-chlorophenyl)-6-ethyl-2,4- pyrimidinediamine} is an antifolate drug and a medication used in combination with other drugs for treatment of protozoan disease like toxoplasmosis, bacterial infections and some types of cancer (Zuccotto *et al.*, 1998; Kraut & Matthews, 1987). Pyrimethamine (PMN) exhibits a donor–acceptor–donor site, so that together with a complimentary molecule it can form three hydrogen bonds, yielding a robust supramolecular synthon (Desiraju, 1995). The crystal structure of 2-amino-4,6-dimethylpyrimidine-cinnamic acid (Balasubramani *et al.*, 2005), pyrimethaminium 3,5-dinitrobenzoate (Subashini *et al.*, 2007),pyrimethamine hydrogen adipate (Devi *et al.*, 2007),2-amino-4,6-dimethylpyrimidine-terephthalic acid (Devi *et al.*, 2006), 2-amino-4,6-dimethylpyrimidine-anthranilic acid (Ebenezer & Muthiah , 2010), 2-amino-4,6-dimethoxypyrimidinium picrate and pyrimethaminium picrate dimethyl sulfoxide solvate (Thanigaimani *et al.*, 2009) have been reported. Recently, the structure of [2-(4- (Trifluoromethyl)phenylsulfanyl]benzoic acid (Yamuna *et al.*, 2013) used in the preparation of the title compound was reported by our research group. As part of our studies in this area, this paper reports the crystal structure of the title compound, (I), (Fig. 1).

In the cation, the dihedral angle between the mean plane of the pyrimidinium and the 4-chlorophenyl ring is 77.2 (5)°. In the anion, the mean planes of the two phenyl rings are twisted with respect to each other by 71.5 (5)°. Disorder was modelled for the dimethyl sulfoxide solvent molecule over two sites in a 0.7487 (13):0.2513 (13) ratio. Within the asymmetric unit, cation-anion N—H···O hydrogen bonds (forming R_2_^2^(8) graph-set ring motifs) along with cation-cation N—H···N hydrogen bonds are observed. In the crystal additional cation-cation N—H···N hydrogen bonds and cation-solvate N—H···O hydrogen bonds help to consolidate the packing (Fig. 2). Weak cation-anion C—H···F, and solvate-anion C—H···O, C—H···F are also observed (Table 1).

### 2. Experimental

Pyrimethamine (0.5 g, 0.2010 mmol) and 2-(4-trifluoromethylphenyl sulfanyl)benzoic acid(0.599 g, 0.2010 mmol) were dissolved in 10 ml of hot dimethyl sulphoxide solution and stirred for 20 minutes and kept aside for slow evaporation. After few days, irregular colourless chunks of the title compound were developed (m.p: 383–388 K).

### 3. Refinement

All of the H atoms were placed in their calculated positions and then refined using the riding model with Atom—H lengths of 0.95Å (CH); 0.99Å (CH_2_); 0.98Å (CH_3_) or 0.88Å (NH, NH_2_). Isotropic displacement parameters for these atoms were set to 1.2 (CH, CH_2_, NH, NH_2_) or 1.5 (CH_3_) times *U*_eq_ of the parent atom. Idealised Me groups refined as rotating groups. Disorder was modelled for the S1S, O1S, C1S and C2S atoms of the dimethyl sulfoxide solvent molecule over two sites in a 0.7487 (13):0.2513 (13) ratio.

### Crystal data

**Table:**

C_12_H_14_ClN_4_^+^·C_14_H_8_F_3_O_2_S^−^·C_2_H_6_OS	*F*(000) = 1296
*M**_r_* = 625.11	*D*_x_ = 1.433 Mg m^−^^3^
Monoclinic, *P*2_1_/*c*	Cu *K*α radiation, λ = 1.54184 Å
*a* = 12.7422 (3) Å	Cell parameters from 8424 reflections
*b* = 22.2773 (3) Å	θ = 4.0–71.5°
*c* = 11.1761 (3) Å	µ = 3.01 mm^−^^1^
β = 114.014 (3)°	*T* = 173 K
*V* = 2897.88 (12) Å^3^	Irregular, colourless
*Z* = 4	0.36 × 0.18 × 0.06 mm

### Data collection

**Table:**

Agilent Eos Gemini diffractometer	5571 independent reflections
Radiation source: Enhance (Cu) X-ray Source	4889 reflections with *I* > 2σ(*I*)
Detector resolution: 16.0416 pixels mm^-1^	*R*_int_ = 0.046
ω scans	θ_max_ = 71.4°, θ_min_ = 3.8°
Absorption correction: multi-scan (*CrysAlis RED*; Agilent, 2012)	*h* = −15→15
*T*_min_ = 0.374, *T*_max_ = 1.000	*k* = −27→27
19462 measured reflections	*l* = −8→13

### Refinement

**Table:**

Refinement on *F*^2^	Hydrogen site location: inferred from neighbouring sites
Least-squares matrix: full	H-atom parameters constrained
*R*[*F*^2^ > 2σ(*F*^2^)] = 0.049	*w* = 1/[σ^2^(*F*_o_^2^) + (0.0872*P*)^2^ + 1.2456*P*] where *P* = (*F*_o_^2^ + 2*F*_c_^2^)/3
*wR*(*F*^2^) = 0.138	(Δ/σ)_max_ < 0.001
*S* = 1.02	Δρ_max_ = 0.72 e Å^−^^3^
5571 reflections	Δρ_min_ = −0.41 e Å^−^^3^
389 parameters	Extinction correction: *SHELXL2012* (Sheldrick, 2008), Fc^*^=kFc[1+0.001xFc^2^λ^3^/sin(2θ)]^-1/4^
54 restraints	Extinction coefficient: 0.00062 (19)
Primary atom site location: structure-invariant direct methods	

### Special details

**Table:**

Geometry. All esds (except the esd in the dihedral angle between two l.s. planes) are estimated using the full covariance matrix. The cell esds are taken into account individually in the estimation of esds in distances, angles and torsion angles; correlations between esds in cell parameters are only used when they are defined by crystal symmetry. An approximate (isotropic) treatment of cell esds is used for estimating esds involving l.s. planes.

### Fractional atomic coordinates and isotropic or equivalent isotropic displacement parameters (Å^2^)

**Table:**

	*x*	*y*	*z*	*U*_iso_*/*U*_eq_	Occ. (<1)
S1	0.59857 (4)	0.47670 (2)	0.25721 (5)	0.02905 (16)	
F1	0.88133 (16)	0.63579 (7)	−0.0149 (2)	0.0652 (5)	
F2	0.85792 (17)	0.55628 (11)	−0.13276 (16)	0.0758 (6)	
F3	1.00185 (12)	0.56365 (7)	0.05033 (16)	0.0488 (4)	
O1	0.45576 (13)	0.44517 (6)	0.36254 (16)	0.0343 (3)	
O2	0.40640 (15)	0.35336 (7)	0.40052 (19)	0.0431 (4)	
C13	0.45497 (17)	0.38898 (9)	0.3526 (2)	0.0304 (4)	
C14	0.51534 (17)	0.36331 (9)	0.2722 (2)	0.0311 (4)	
C14A	0.8891 (2)	0.57575 (11)	−0.0105 (2)	0.0410 (5)	
C15	0.57766 (16)	0.39873 (9)	0.2191 (2)	0.0280 (4)	
C16	0.61951 (18)	0.37158 (10)	0.1337 (2)	0.0356 (5)	
H16	0.6614	0.3949	0.0969	0.043*	
C17	0.6006 (2)	0.31154 (12)	0.1027 (3)	0.0504 (7)	
H17	0.6282	0.2942	0.0434	0.060*	
C18	0.5418 (2)	0.27616 (11)	0.1570 (4)	0.0593 (8)	
H18	0.5297	0.2346	0.1365	0.071*	
C19	0.5010 (2)	0.30267 (11)	0.2418 (3)	0.0466 (6)	
H19	0.4618	0.2785	0.2806	0.056*	
C20	0.68608 (17)	0.49999 (9)	0.17504 (19)	0.0277 (4)	
C21	0.63817 (18)	0.53953 (10)	0.0700 (2)	0.0332 (4)	
H21	0.5591	0.5497	0.0388	0.040*	
C22	0.7044 (2)	0.56394 (10)	0.0109 (2)	0.0373 (5)	
H22	0.6714	0.5911	−0.0602	0.045*	
C23	0.81971 (19)	0.54862 (9)	0.0557 (2)	0.0314 (4)	
C24	0.86825 (18)	0.50883 (10)	0.1591 (2)	0.0330 (4)	
H24	0.9469	0.4979	0.1886	0.040*	
C25	0.80155 (18)	0.48508 (10)	0.2193 (2)	0.0316 (4)	
H25	0.8350	0.4584	0.2912	0.038*	
Cl1	0.08126 (6)	0.85545 (2)	0.33795 (7)	0.0507 (2)	
N1	0.28497 (13)	0.50285 (7)	0.39467 (15)	0.0239 (3)	
H1	0.3410	0.4845	0.3824	0.029*	
N2	0.12544 (14)	0.49511 (7)	0.44931 (16)	0.0257 (3)	
N3	0.22872 (16)	0.41131 (8)	0.44299 (19)	0.0341 (4)	
H3A	0.1826	0.3888	0.4650	0.041*	
H3B	0.2859	0.3947	0.4300	0.041*	
N4	0.02748 (15)	0.57911 (8)	0.45960 (18)	0.0303 (4)	
H4A	−0.0176	0.5560	0.4819	0.036*	
H4B	0.0160	0.6181	0.4525	0.036*	
C1	0.21210 (16)	0.47019 (8)	0.42950 (18)	0.0243 (4)	
C2	0.11292 (16)	0.55510 (8)	0.43659 (18)	0.0243 (4)	
C3	0.18716 (16)	0.59204 (8)	0.39895 (18)	0.0241 (4)	
C4	0.27322 (16)	0.56345 (8)	0.37826 (17)	0.0234 (4)	
C5	0.35677 (18)	0.59368 (9)	0.3344 (2)	0.0288 (4)	
H5A	0.4349	0.5780	0.3865	0.035*	
H5B	0.3572	0.6373	0.3514	0.035*	
C6	0.3271 (2)	0.58358 (11)	0.1887 (2)	0.0398 (5)	
H6A	0.3299	0.5405	0.1721	0.060*	
H6B	0.3827	0.6049	0.1641	0.060*	
H6C	0.2497	0.5989	0.1367	0.060*	
C7	0.16593 (16)	0.65788 (8)	0.38331 (19)	0.0248 (4)	
C8	0.19577 (19)	0.69472 (9)	0.4931 (2)	0.0311 (4)	
H8	0.2334	0.6780	0.5782	0.037*	
C9	0.1710 (2)	0.75557 (9)	0.4791 (2)	0.0350 (5)	
H9	0.1923	0.7806	0.5541	0.042*	
C10	0.11506 (18)	0.77934 (9)	0.3554 (2)	0.0326 (5)	
C11	0.08432 (19)	0.74404 (10)	0.2442 (2)	0.0338 (5)	
H11	0.0462	0.7610	0.1593	0.041*	
C12	0.11064 (18)	0.68325 (9)	0.2598 (2)	0.0297 (4)	
H12	0.0904	0.6585	0.1845	0.036*	
S1SA	0.77732 (6)	0.71883 (3)	0.36675 (8)	0.0390 (2)	0.7487 (13)
O1SA	0.8835 (4)	0.68442 (14)	0.4534 (4)	0.0415 (8)	0.7487 (13)
C1SA	0.6760 (7)	0.6652 (8)	0.2710 (19)	0.0757 (14)	0.7487 (13)
H1SA	0.6414	0.6455	0.3246	0.114*	0.7487 (13)
H1SB	0.7144	0.6351	0.2389	0.114*	0.7487 (13)
H1SC	0.6159	0.6852	0.1965	0.114*	0.7487 (13)
C2SA	0.8085 (6)	0.7538 (3)	0.2407 (7)	0.0677 (16)	0.7487 (13)
H2SA	0.8725	0.7819	0.2803	0.102*	0.7487 (13)
H2SB	0.7405	0.7755	0.1809	0.102*	0.7487 (13)
H2SC	0.8297	0.7230	0.1919	0.102*	0.7487 (13)
S1SB	0.80800 (19)	0.68308 (10)	0.2873 (2)	0.0390 (2)	0.2513 (13)
O1SB	0.8765 (16)	0.6714 (6)	0.4284 (13)	0.0415 (8)	0.2513 (13)
C1SB	0.664 (2)	0.667 (3)	0.260 (6)	0.0757 (14)	0.2513 (13)
H1SD	0.6569	0.6245	0.2787	0.114*	0.2513 (13)
H1SE	0.6132	0.6754	0.1683	0.114*	0.2513 (13)
H1SF	0.6409	0.6919	0.3175	0.114*	0.2513 (13)
C2SB	0.794 (2)	0.7625 (7)	0.270 (3)	0.0677 (16)	0.2513 (13)
H2SD	0.8687	0.7799	0.2820	0.102*	0.2513 (13)
H2SE	0.7697	0.7788	0.3355	0.102*	0.2513 (13)
H2SF	0.7372	0.7723	0.1819	0.102*	0.2513 (13)

### Atomic displacement parameters (Å^2^)

**Table:**

	*U*^11^	*U*^22^	*U*^33^	*U*^12^	*U*^13^	*U*^23^
S1	0.0340 (3)	0.0260 (3)	0.0308 (3)	0.00097 (17)	0.0168 (2)	−0.00265 (17)
F1	0.0647 (11)	0.0439 (9)	0.0959 (14)	−0.0030 (7)	0.0418 (10)	0.0216 (8)
F2	0.0761 (12)	0.1208 (17)	0.0363 (9)	−0.0481 (12)	0.0287 (8)	−0.0166 (9)
F3	0.0403 (8)	0.0504 (8)	0.0605 (9)	−0.0097 (6)	0.0254 (7)	−0.0024 (7)
O1	0.0374 (8)	0.0294 (7)	0.0446 (9)	0.0013 (6)	0.0254 (7)	−0.0030 (6)
O2	0.0435 (9)	0.0340 (8)	0.0627 (11)	−0.0035 (7)	0.0327 (8)	0.0002 (7)
C13	0.0246 (9)	0.0316 (10)	0.0342 (10)	0.0011 (8)	0.0113 (8)	−0.0016 (8)
C14	0.0214 (9)	0.0295 (10)	0.0404 (11)	0.0033 (7)	0.0104 (8)	−0.0022 (8)
C14A	0.0433 (13)	0.0408 (12)	0.0387 (12)	−0.0119 (10)	0.0165 (10)	−0.0029 (9)
C15	0.0216 (9)	0.0274 (9)	0.0315 (10)	0.0038 (7)	0.0070 (8)	−0.0039 (7)
C16	0.0268 (10)	0.0390 (11)	0.0427 (12)	0.0001 (8)	0.0158 (9)	−0.0111 (9)
C17	0.0368 (12)	0.0470 (14)	0.0759 (18)	−0.0015 (10)	0.0316 (13)	−0.0260 (13)
C18	0.0477 (14)	0.0316 (12)	0.113 (3)	−0.0061 (11)	0.0467 (16)	−0.0258 (14)
C19	0.0344 (12)	0.0321 (11)	0.0814 (19)	−0.0024 (9)	0.0319 (12)	−0.0079 (11)
C20	0.0308 (10)	0.0260 (9)	0.0264 (10)	−0.0011 (7)	0.0117 (8)	−0.0030 (7)
C21	0.0288 (10)	0.0332 (10)	0.0326 (11)	0.0002 (8)	0.0074 (8)	0.0022 (8)
C22	0.0373 (12)	0.0365 (11)	0.0302 (11)	−0.0028 (9)	0.0057 (9)	0.0069 (8)
C23	0.0346 (11)	0.0302 (10)	0.0272 (10)	−0.0089 (8)	0.0105 (8)	−0.0066 (8)
C24	0.0284 (10)	0.0376 (11)	0.0312 (10)	0.0006 (8)	0.0102 (8)	−0.0032 (8)
C25	0.0330 (11)	0.0343 (10)	0.0259 (10)	0.0050 (8)	0.0104 (8)	0.0032 (8)
Cl1	0.0609 (4)	0.0217 (3)	0.0834 (5)	0.0048 (2)	0.0435 (4)	−0.0001 (2)
N1	0.0252 (8)	0.0232 (8)	0.0268 (8)	−0.0005 (6)	0.0143 (6)	−0.0013 (6)
N2	0.0249 (8)	0.0244 (8)	0.0302 (8)	−0.0024 (6)	0.0138 (7)	0.0021 (6)
N3	0.0383 (10)	0.0228 (8)	0.0514 (11)	0.0008 (7)	0.0289 (9)	0.0049 (7)
N4	0.0288 (8)	0.0240 (8)	0.0451 (10)	0.0000 (6)	0.0222 (8)	0.0037 (7)
C1	0.0251 (9)	0.0238 (9)	0.0241 (9)	−0.0021 (7)	0.0101 (7)	−0.0006 (7)
C2	0.0241 (9)	0.0252 (9)	0.0234 (9)	−0.0029 (7)	0.0096 (7)	0.0004 (7)
C3	0.0259 (9)	0.0243 (9)	0.0228 (9)	−0.0029 (7)	0.0105 (7)	0.0002 (7)
C4	0.0253 (9)	0.0252 (9)	0.0208 (9)	−0.0046 (7)	0.0105 (7)	−0.0023 (6)
C5	0.0305 (10)	0.0272 (9)	0.0349 (11)	−0.0057 (7)	0.0197 (9)	−0.0018 (7)
C6	0.0437 (13)	0.0488 (13)	0.0344 (12)	−0.0031 (10)	0.0235 (10)	0.0052 (9)
C7	0.0244 (9)	0.0228 (9)	0.0323 (10)	−0.0036 (7)	0.0167 (8)	−0.0012 (7)
C8	0.0369 (11)	0.0303 (10)	0.0302 (10)	−0.0063 (8)	0.0177 (9)	−0.0022 (8)
C9	0.0453 (12)	0.0272 (10)	0.0418 (12)	−0.0095 (9)	0.0272 (10)	−0.0111 (8)
C10	0.0351 (11)	0.0208 (9)	0.0528 (13)	−0.0014 (8)	0.0289 (10)	−0.0015 (8)
C11	0.0378 (11)	0.0279 (10)	0.0385 (11)	0.0044 (8)	0.0185 (9)	0.0061 (8)
C12	0.0361 (11)	0.0249 (9)	0.0302 (10)	−0.0002 (8)	0.0155 (8)	−0.0032 (7)
S1SA	0.0363 (4)	0.0300 (3)	0.0499 (4)	0.0043 (3)	0.0168 (3)	0.0011 (3)
O1SA	0.0374 (12)	0.0271 (19)	0.0538 (19)	0.0023 (15)	0.0121 (14)	0.0044 (13)
C1SA	0.067 (3)	0.0428 (19)	0.078 (4)	−0.012 (2)	−0.011 (3)	−0.003 (2)
C2SA	0.063 (3)	0.073 (3)	0.080 (4)	0.018 (2)	0.041 (2)	0.033 (3)
S1SB	0.0363 (4)	0.0300 (3)	0.0499 (4)	0.0043 (3)	0.0168 (3)	0.0011 (3)
O1SB	0.0374 (12)	0.0271 (19)	0.0538 (19)	0.0023 (15)	0.0121 (14)	0.0044 (13)
C1SB	0.067 (3)	0.0428 (19)	0.078 (4)	−0.012 (2)	−0.011 (3)	−0.003 (2)
C2SB	0.063 (3)	0.073 (3)	0.080 (4)	0.018 (2)	0.041 (2)	0.033 (3)

### Geometric parameters (Å, º)

**Table:**

S1—C15	1.782 (2)	N4—C2	1.329 (3)
S1—C20	1.786 (2)	C2—C3	1.440 (3)
F1—C14A	1.341 (3)	C3—C4	1.367 (3)
F2—C14A	1.331 (3)	C3—C7	1.489 (3)
F3—C14A	1.343 (3)	C4—C5	1.501 (3)
O1—C13	1.256 (3)	C5—H5A	0.9900
O2—C13	1.252 (3)	C5—H5B	0.9900
C13—C14	1.514 (3)	C5—C6	1.531 (3)
C14—C15	1.410 (3)	C6—H6A	0.9800
C14—C19	1.387 (3)	C6—H6B	0.9800
C14A—C23	1.492 (3)	C6—H6C	0.9800
C15—C16	1.405 (3)	C7—C8	1.394 (3)
C16—H16	0.9500	C7—C12	1.389 (3)
C16—C17	1.378 (3)	C8—H8	0.9500
C17—H17	0.9500	C8—C9	1.386 (3)
C17—C18	1.386 (4)	C9—H9	0.9500
C18—H18	0.9500	C9—C10	1.378 (3)
C18—C19	1.385 (4)	C10—C11	1.386 (3)
C19—H19	0.9500	C11—H11	0.9500
C20—C21	1.394 (3)	C11—C12	1.389 (3)
C20—C25	1.388 (3)	C12—H12	0.9500
C21—H21	0.9500	S1SA—O1SA	1.513 (4)
C21—C22	1.378 (3)	S1SA—C1SA	1.765 (14)
C22—H22	0.9500	S1SA—C2SA	1.791 (5)
C22—C23	1.388 (3)	C1SA—H1SA	0.9800
C23—C24	1.387 (3)	C1SA—H1SB	0.9800
C24—H24	0.9500	C1SA—H1SC	0.9800
C24—C25	1.385 (3)	C2SA—H2SA	0.9800
C25—H25	0.9500	C2SA—H2SB	0.9800
Cl1—C10	1.741 (2)	C2SA—H2SC	0.9800
N1—H1	0.8800	S1SB—O1SB	1.482 (13)
N1—C1	1.356 (2)	S1SB—C1SB	1.78 (2)
N1—C4	1.362 (2)	S1SB—C2SB	1.780 (15)
N2—C1	1.332 (3)	C1SB—H1SD	0.9800
N2—C2	1.347 (2)	C1SB—H1SE	0.9800
N3—H3A	0.8800	C1SB—H1SF	0.9800
N3—H3B	0.8800	C2SB—H2SD	0.9800
N3—C1	1.327 (3)	C2SB—H2SE	0.9800
N4—H4A	0.8800	C2SB—H2SF	0.9800
N4—H4B	0.8800		
			
C15—S1—C20	102.95 (9)	C4—C3—C7	124.00 (17)
O1—C13—C14	116.17 (18)	N1—C4—C3	119.40 (17)
O2—C13—O1	125.6 (2)	N1—C4—C5	115.78 (17)
O2—C13—C14	118.25 (19)	C3—C4—C5	124.80 (17)
C15—C14—C13	123.22 (18)	C4—C5—H5A	109.1
C19—C14—C13	117.7 (2)	C4—C5—H5B	109.1
C19—C14—C15	118.9 (2)	C4—C5—C6	112.35 (16)
F1—C14A—F3	105.48 (19)	H5A—C5—H5B	107.9
F1—C14A—C23	111.9 (2)	C6—C5—H5A	109.1
F2—C14A—F1	107.5 (2)	C6—C5—H5B	109.1
F2—C14A—F3	105.4 (2)	C5—C6—H6A	109.5
F2—C14A—C23	112.77 (19)	C5—C6—H6B	109.5
F3—C14A—C23	113.3 (2)	C5—C6—H6C	109.5
C14—C15—S1	119.98 (15)	H6A—C6—H6B	109.5
C16—C15—S1	121.39 (17)	H6A—C6—H6C	109.5
C16—C15—C14	118.62 (19)	H6B—C6—H6C	109.5
C15—C16—H16	119.6	C8—C7—C3	120.35 (18)
C17—C16—C15	120.9 (2)	C12—C7—C3	120.80 (17)
C17—C16—H16	119.6	C12—C7—C8	118.76 (18)
C16—C17—H17	119.6	C7—C8—H8	119.7
C16—C17—C18	120.8 (2)	C9—C8—C7	120.6 (2)
C18—C17—H17	119.6	C9—C8—H8	119.7
C17—C18—H18	120.7	C8—C9—H9	120.3
C19—C18—C17	118.5 (2)	C10—C9—C8	119.33 (19)
C19—C18—H18	120.7	C10—C9—H9	120.3
C14—C19—H19	118.9	C9—C10—Cl1	119.36 (17)
C18—C19—C14	122.2 (2)	C9—C10—C11	121.61 (19)
C18—C19—H19	118.9	C11—C10—Cl1	119.03 (18)
C21—C20—S1	117.75 (16)	C10—C11—H11	120.8
C25—C20—S1	122.71 (16)	C10—C11—C12	118.3 (2)
C25—C20—C21	119.23 (19)	C12—C11—H11	120.8
C20—C21—H21	119.7	C7—C12—C11	121.40 (19)
C22—C21—C20	120.6 (2)	C7—C12—H12	119.3
C22—C21—H21	119.7	C11—C12—H12	119.3
C21—C22—H22	120.2	O1SA—S1SA—C1SA	106.8 (5)
C21—C22—C23	119.7 (2)	O1SA—S1SA—C2SA	107.3 (3)
C23—C22—H22	120.2	C1SA—S1SA—C2SA	99.2 (7)
C22—C23—C14A	118.4 (2)	S1SA—C1SA—H1SA	109.5
C24—C23—C14A	121.3 (2)	S1SA—C1SA—H1SB	109.5
C24—C23—C22	120.3 (2)	S1SA—C1SA—H1SC	109.5
C23—C24—H24	120.2	H1SA—C1SA—H1SB	109.5
C25—C24—C23	119.7 (2)	H1SA—C1SA—H1SC	109.5
C25—C24—H24	120.2	H1SB—C1SA—H1SC	109.5
C20—C25—H25	119.8	S1SA—C2SA—H2SA	109.5
C24—C25—C20	120.43 (19)	S1SA—C2SA—H2SB	109.5
C24—C25—H25	119.8	S1SA—C2SA—H2SC	109.5
C1—N1—H1	119.3	H2SA—C2SA—H2SB	109.5
C1—N1—C4	121.32 (16)	H2SA—C2SA—H2SC	109.5
C4—N1—H1	119.3	H2SB—C2SA—H2SC	109.5
C1—N2—C2	117.81 (16)	O1SB—S1SB—C1SB	105.6 (19)
H3A—N3—H3B	120.0	O1SB—S1SB—C2SB	106.3 (10)
C1—N3—H3A	120.0	C1SB—S1SB—C2SB	97.8 (18)
C1—N3—H3B	120.0	S1SB—C1SB—H1SD	109.5
H4A—N4—H4B	120.0	S1SB—C1SB—H1SE	109.5
C2—N4—H4A	120.0	S1SB—C1SB—H1SF	109.5
C2—N4—H4B	120.0	H1SD—C1SB—H1SE	109.5
N2—C1—N1	122.34 (17)	H1SD—C1SB—H1SF	109.5
N3—C1—N1	117.82 (17)	H1SE—C1SB—H1SF	109.5
N3—C1—N2	119.84 (17)	S1SB—C2SB—H2SD	109.5
N2—C2—C3	122.31 (17)	S1SB—C2SB—H2SE	109.5
N4—C2—N2	116.76 (17)	S1SB—C2SB—H2SF	109.5
N4—C2—C3	120.92 (17)	H2SD—C2SB—H2SE	109.5
C2—C3—C7	119.20 (17)	H2SD—C2SB—H2SF	109.5
C4—C3—C2	116.79 (17)	H2SE—C2SB—H2SF	109.5
			
S1—C15—C16—C17	−178.56 (19)	C25—C20—C21—C22	−0.4 (3)
S1—C20—C21—C22	173.43 (17)	Cl1—C10—C11—C12	−179.21 (16)
S1—C20—C25—C24	−173.98 (16)	N1—C4—C5—C6	76.0 (2)
F1—C14A—C23—C22	53.3 (3)	N2—C2—C3—C4	−1.0 (3)
F1—C14A—C23—C24	−126.9 (2)	N2—C2—C3—C7	178.32 (17)
F2—C14A—C23—C22	−68.0 (3)	N4—C2—C3—C4	179.53 (18)
F2—C14A—C23—C24	111.8 (3)	N4—C2—C3—C7	−1.2 (3)
F3—C14A—C23—C22	172.42 (19)	C1—N1—C4—C3	0.9 (3)
F3—C14A—C23—C24	−7.8 (3)	C1—N1—C4—C5	−177.64 (17)
O1—C13—C14—C15	5.1 (3)	C1—N2—C2—N4	−178.57 (18)
O1—C13—C14—C19	−170.1 (2)	C1—N2—C2—C3	1.9 (3)
O2—C13—C14—C15	−176.7 (2)	C2—N2—C1—N1	−1.5 (3)
O2—C13—C14—C19	8.1 (3)	C2—N2—C1—N3	178.96 (18)
C13—C14—C15—S1	5.4 (3)	C2—C3—C4—N1	−0.4 (3)
C13—C14—C15—C16	−172.93 (19)	C2—C3—C4—C5	177.92 (17)
C13—C14—C19—C18	172.7 (2)	C2—C3—C7—C8	75.3 (2)
C14—C15—C16—C17	−0.3 (3)	C2—C3—C7—C12	−101.2 (2)
C14A—C23—C24—C25	179.2 (2)	C3—C4—C5—C6	−102.4 (2)
C15—S1—C20—C21	113.10 (17)	C3—C7—C8—C9	−176.74 (19)
C15—S1—C20—C25	−73.32 (19)	C3—C7—C12—C11	176.20 (19)
C15—C14—C19—C18	−2.6 (4)	C4—N1—C1—N2	0.1 (3)
C15—C16—C17—C18	−1.3 (4)	C4—N1—C1—N3	179.68 (17)
C16—C17—C18—C19	0.9 (5)	C4—C3—C7—C8	−105.4 (2)
C17—C18—C19—C14	1.1 (5)	C4—C3—C7—C12	78.1 (3)
C19—C14—C15—S1	−179.52 (18)	C7—C3—C4—N1	−179.68 (17)
C19—C14—C15—C16	2.2 (3)	C7—C3—C4—C5	−1.3 (3)
C20—S1—C15—C14	177.34 (16)	C7—C8—C9—C10	0.8 (3)
C20—S1—C15—C16	−4.38 (19)	C8—C7—C12—C11	−0.4 (3)
C20—C21—C22—C23	0.5 (3)	C8—C9—C10—Cl1	178.69 (16)
C21—C20—C25—C24	−0.5 (3)	C8—C9—C10—C11	−0.9 (3)
C21—C22—C23—C14A	179.9 (2)	C9—C10—C11—C12	0.4 (3)
C21—C22—C23—C24	0.2 (3)	C10—C11—C12—C7	0.2 (3)
C22—C23—C24—C25	−1.0 (3)	C12—C7—C8—C9	−0.2 (3)
C23—C24—C25—C20	1.2 (3)		

### Hydrogen-bond geometry (Å, º)

**Table:**

*D*—H···*A*	*D*—H	H···*A*	*D*···*A*	*D*—H···*A*
N1—H1···O1	0.88	1.79	2.674 (2)	178
N3—H3*A*···O1*SA*^i^	0.88	2.20	3.046 (6)	162
N3—H3*A*···O1*SB*^i^	0.88	2.13	2.97 (2)	161
N3—H3*B*···O2	0.88	1.93	2.809 (2)	176
N4—H4*A*···N2^ii^	0.88	2.15	3.030 (2)	175
N4—H4*B*···O1*SA*^iii^	0.88	2.25	2.962 (4)	138
N4—H4*B*···O1*SB*^iii^	0.88	2.06	2.740 (16)	133
C12—H12···F3^iii^	0.95	2.57	3.444 (2)	153
C2*SA*—H2*SB*···O2^iv^	0.98	2.44	3.376 (6)	160
C2*SB*—H2*SE*···F1^v^	0.98	2.55	3.16 (3)	120
C2*SB*—H2*SF*···O2^iv^	0.98	2.47	3.21 (2)	132

Symmetry codes: (i) −*x*+1, −*y*+1, −*z*+1; (ii) −*x*, −*y*+1, −*z*+1; (iii) *x*−1, *y*, *z*; (iv) −*x*+1, *y*+1/2, −*z*+1/2; (v) *x*, −*y*+3/2, *z*+1/2.
